# Interleukin-6, anxiety, and pre-treatment malnutrition in locally advanced head and neck cancer: a psychoneuroimmunological analysis

**DOI:** 10.3389/fnut.2026.1918262

**Published:** 2026-08-11

**Authors:** Lucía Aránega-Martín, Lidia Sánchez-Alcoholado, Alicia González-González, María Dolores Toledo-Serrano, María Jesús García-Anaya, Isaac Plaza-Andrades, Rocío Fernández-Jiménez, José Manuel García-Almeida, María Jesús Lorca-Ocón, María Isabel Queipo-Ortuño, Jaime Gómez-Millán

**Affiliations:** 1 Medical Oncology Clinical Management Unit, Virgen de la Victoria University Hospital, Málaga, Spain; 2 Málaga Biomedical Research Institute (IBIMA)-BIONAND Platform, Málaga, Spain; 3 Department of Surgical Specialties, Biochemical and Immunology, Faculty of Medicine, University of Málaga, Málaga, Spain; 4 Obesity, Diabetes and Metabolism Laboratory, Biomedical Research Institute of Murcia (IMIB), Murcia, Spain; 5 Department of Endocrinology and Nutrition, Hospital Regional Universitario de Málaga, Instituto de Investigación Biomédica de Málaga y Plataforma en Nanomedicina (IBIMA-Plataforma BIONAND), Málaga, Spain; 6 Radiation Oncology Unit, Virgen de la Victoria University Hospital, Málaga, Spain; 7 Endocrinology and Nutrition Unit, Virgen de la Victoria University Hospital, Málaga, Spain; 8 Department of Endocrinology and Nutrition, Quirón Salud Málaga Hospital, Málaga, Spain

**Keywords:** anxiety, GLIM criteria, head and neck cancer, interleukin-6, malnutrition, psychoneuroimmunology, quality of life

## Abstract

**Background:**

Malnutrition and psychological distress are highly prevalent in patients with locally advanced head and neck cancer (LAHNC), although the biological mechanisms linking these conditions remain poorly understood. We investigated associations between systemic inflammation, gut–brain axis hormones, anxiety, and pre-treatment malnutrition within a psychoneuroimmunological framework.

**Methods:**

A cross-sectional study was conducted in 74 patients with LAHNC prior to treatment initiation. Nutritional status was assessed using the Global Leadership Initiative on Malnutrition (GLIM) criteria, and anxiety using the Hospital Anxiety and Depression Scale (HADS-A). Circulating inflammatory cytokines and appetite-regulating hormones were quantified using multiplex immunoassays. Multivariable logistic regression and exploratory bootstrap mediation analyses were performed, together with an exploratory, bootstrap-based mediation analysis to evaluate whether anxiety represented a potential indirect pathway linking IL-6 and pre-treatment malnutrition.

**Results:**

Pre-treatment malnutrition was identified in 40.5% of patients and clinically relevant anxiety in 36.5%. Elevated IL-6 and lower BMI were independently associated with malnutrition (OR 6.16, 95% CI 1.35–28.18; and OR 0.83, 95% CI 0.72–0.97, respectively). Elevated IL-6 was also independently associated with anxiety (OR 11.23, 95% CI 2.09–60.40). Elevated glucose-dependent insulinotropic polypeptide (GIP) levels were also associated with malnutrition after false discovery rate correction (*q* = 0.003). The exploratory mediation analysis suggested a non-significant trend toward partial mediation of the IL-6-malnutrition association through anxiety (the indirect effect did not reach statistical significance). Patients with malnutrition and anxiety exhibited significantly impaired health-related quality of life.

**Conclusion:**

These findings are consistent with a psychoneuroimmunological framework linking IL-6, anxiety, and pre-treatment malnutrition in LAHNC. Integrative strategies targeting inflammatory burden, nutritional status, and psychological distress may help improve clinical outcomes in this population.

## 1 Introduction

Malnutrition is one of the most prevalent and clinically significant complications in patients with locally advanced head and neck cancer (LAHNC), with rates exceeding 50% at diagnosis and increasing substantially during radiotherapy or concurrent chemoradiotherapy (RCT) (1, 2). According to the Global Leadership Initiative on Malnutrition (GLIM) criteria, its diagnosis requires at least one phenotypic criterion (unintentional weight loss, low BMI, or reduced muscle mass) and at least one etiologic criterion, such as reduced food intake or disease-related inflammation (3).

The pathophysiology of malnutrition in LAHNC is multifactorial (4). Dysphagia, odynophagia, mucositis, taste alterations, and pain compromise oral intake even before treatment initiation (5, 6), while pre-treatment malnutrition has been independently associated with reduced treatment tolerance, increased toxicity, and worse survival (7, 8). Beyond these mechanical and treatment-related factors, psychological distress, which is highly prevalent in head and neck cancer, plays a pivotal and underexplored role. Evidence from psychoneuroimmunology (PNI) demonstrates that emotional distress activates the hypothalamic–pituitary–adrenal (HPA) axis and the sympathetic nervous system, promoting hypersecretion of glucocorticoids and catecholamines that sustain systemic inflammation, characterised by elevated IL-6 and TNF-α (9, 10). These cytokines directly drive anorexia, muscle catabolism, and cancer cachexia; notably, IL-6 acts on the area postrema of the brainstem to dysregulate appetite and energy expenditure (11, 12).

The gut–brain axis further mediates this relationship through bidirectional neuroimmune signaling. Gut-derived hormones that regulate this axis, including ghrelin, leptin, peptide YY (PYY), glucose-dependent insulinotropic polypeptide (GIP), and glucagon-like peptide-1 (GLP-1) are key regulators of appetite and energy homeostasis whose dysregulation has been implicated in cancer-related anorexia and cachexia (13–16). Psychological distress and systemic inflammation may synergistically disrupt these hormonal signals, amplifying nutritional decline beyond the contribution of tumour burden or treatment toxicity alone.

Although associations between psychological distress, assessed by the Hospital Anxiety and Depression Scale (HADS), and impaired nutritional status have been previously reported (17–19), the biological mediators underlying this relationship in LAHNC remain poorly characterised.

Human papillomavirus (HPV)-associated oropharyngeal carcinomas represent a biologically distinct subgroup of head and neck cancer, characterised by different epidemiological, inflammatory and clinical features, including improved treatment response and prognosis. Because these differences may influence nutritional status and clinical outcomes, HPV status was considered as a potential confounding variable in the present study (20, 21).

We hypothesized that elevated inflammatory cytokines and gut-derived appetite-regulating hormones would be independently associated with both anxiety and pre-treatment malnutrition and explored whether anxiety might partially mediate the relationship between inflammatory burden and nutritional status in this population.

The present study therefore aimed to evaluate the impact of psychological distress on nutritional status, defined by GLIM criteria, in patients with LAHNC who were scheduled to receive definitive RCT (22), investigating the role of circulating pro-inflammatory cytokines and gut–brain axis hormones as exploratory mediators. Elucidating these mechanisms may support more integrative nutritional and psycho-oncological care strategies in head and neck cancer.

Accordingly, the primary objective of this study was to investigate whether circulating IL-6 is independently associated with pre-treatment malnutrition in patients with LAHNC. Secondary objectives included evaluating the relationship between IL-6 and anxiety symptoms, as well as health-related quality of life. Finally, based on the psychoneuroimmunological literature, we performed an exploratory mediation analysis to examine whether anxiety could represent a potential indirect pathway linking systemic inflammation with malnutrition. Given the cross-sectional design, this mediation analysis was considered hypothesis-generating rather than confirmatory.

## 2 Materials and methods

### 2.1 Study design, population, and clinical assessments

We conducted a cross-sectional study at the Radiation Oncology Department of the University Hospital Virgen de la Victoria (Málaga, Spain). The study population comprised 74 adults with histologically confirmed locally advanced head and neck squamous cell carcinoma (LAHNC) (stage III–IV) who were scheduled to receive definitive radiotherapy or concurrent chemoradiotherapy (RCT) between 2022 and 2024. Exclusion criteria included prior surgery, radiotherapy, or systemic therapy for any cancer; recurrent or metastatic head and neck disease at baseline; concurrent malignancy; active inflammatory or autoimmune disease, uncontrolled diabetes mellitus, or other clinically significant chronic inflammatory/metabolic comorbidities potentially confounding circulating inflammatory biomarkers; prior initiation of enteral nutritional support (nasogastric or gastrostomy feeding) before the pre-treatment assessment, and incomplete clinical, nutritional, or biochemical data. A total of 85 patients with LAHNC scheduled to receive definitive RCT were initially screened. Of these, 11 patients were excluded, 7 did not meet the strict clinical inclusion/exclusion criteria, and 4 were excluded due to incomplete clinical, nutritional, or biochemical data. Thus, the final sample analyzed consisted of 74 patients. The study was approved by the local Research Ethics Committee, and all participants provided written informed consent in accordance with Declaration of Helsinki.

Demographic and tumour-related variables, including age, sex, tumour site, stage, Eastern Cooperative Oncology Group (ECOG) performance status, and treatment modality, were prospectively recorded. All nutritional assessments, anthropometric measurements, blood sampling, and patient-reported outcome questionnaires were performed during the same pre-treatment clinical visit, before initiation of oncological treatment. Psychological distress was evaluated before treatment initiation using the Hospital Anxiety and Depression Scale (HADS) prior to treatment initiation (23, 24), classifying patients as non-cases (HADS-A score <8) or cases (HADS-A score ≥8), in accordance with validated oncological cut-offs. A cut-off score of ≥8 was selected because it represents the most commonly recommended threshold for identifying clinically relevant anxiety symptoms in oncology populations, providing an appropriate balance between sensitivity and specificity across multiple validation studies. The Hospital Anxiety and Depression Scale (HADS) was specifically developed for use in medically ill populations and is widely recommended for screening psychological distress in oncology. Unlike many generic anxiety questionnaires, the HADS deliberately excludes somatic symptoms such as fatigue, insomnia, appetite loss, or physical weakness, thereby minimizing symptom overlap with cancer- and treatment-related manifestations and improving the specificity of anxiety assessment in patients with chronic medical illnesses. Consequently, the anxiety subscale (HADS-A) has become one of the most extensively validated instruments for assessing anxiety symptoms in patients with cancer, including those with head and neck malignancies. Previous validation studies have consistently demonstrated good psychometric properties for the HADS-A in oncology populations, with Cronbach’s alpha values generally ranging between 0.80 and 0.90, good convergent validity with other standardized anxiety measures, and satisfactory diagnostic accuracy for detecting clinically significant anxiety (25). Item-level HADS-A responses were available for the present sample showing a sample-specific reliability Cronbach’s coefficient of 0.85 (7 items with an item-total correlations ranging from 0.79 to 0.89), calculated from the item-level HADS-A responses in the present sample (*n* = 74). Nutritional status was assessed prior to treatment using the GLIM criteria, which requires the presence of at least one phenotypic and one etiologic criterion for the diagnosis of malnutrition (22, 26–28). Phenotypic criteria include unintentional weight loss of 5–10% within 6 months or 10–20% over a longer period; BMI < 20 kg/m^2^ for individuals younger than 70 years and <22 kg/m^2^ for individuals aged 70 years or older; and reduced muscle mass, as determined by fat-free mass index (FFMI) calculated from fat-free mass (FFM) assessed by bioelectrical impedance analysis (BIA; InBody 770, InBody Co., Seoul, Korea) and adjusted for the patient’s height (Low FFMI thresholds: Men: < 17.0 kg/m^2^ and Women: < 15.0 kg/m^2^). All patients met at least one aetiological criterion, given that cancer is considered a chronic inflammatory condition. Based on GLIM assessment, patients were classified into three nutritional risk categories: normonutrition, moderate malnutrition, or severe malnutrition. For statistical analyses, patients were dichotomised as normonutrition or malnutrition (moderate/ severe). HPV status was obtained from routine pathological reports, determined by p16 immunohistochemistry, and recorded as positive or negative according to institutional diagnostic protocols.

### 2.2 Baseline assessment of circulating gastrointestinal hormones and inflammatory cytokines

Fasting venous blood samples were collected in the morning within 72 h prior to treatment initiation. Serum was obtained by centrifugation at 2,000 × *g* for 10 min, aliquoted, and stored at −80 °C until analysis. Circulating concentrations of gastrointestinal hormones ghrelin, leptin, GIP, GLP-1, PP, PYY, insulin, and C-peptide were quantified simultaneously using the MILLIPLEX® MAP Human Metabolic Hormone Magnetic Bead Panel (cat. No. HMHEMAG-34 K; Merck Life Science S.L., Madrid, Spain), based on Luminex xMAP^®^ technology. Inflammatory cytokines, including the pro-inflammatory cytokines IFN-α2, IL-1α, IL-6, IL-8 and TNF-α, together with the anti-inflammatory cytokine IL-10, were quantified simultaneously using the MILLIPLEX^®^ MAP Human Cytokine/Chemokine Magnetic Bead Panel (cat. No. HCYTOMAG-60 K; Merck Life Science S.L., Madrid, Spain). All samples were measured in duplicate following the manufacturer’s instructions. Analyte concentrations were calculated from standard curves generated for each assay run. Quality control was assessed using kit-supplied internal controls at two concentration levels. Acceptable assay performance was defined as intra-assay coefficient of variation (CV) < 10% and inter-assay CV < 15% for the metabolic hormone panel, and intra-assay CV < 10% and inter-assay CV < 20% for the cytokine panel.

### 2.3 Assessment of health-related quality of life

Health-related quality of life (HRQoL) was assessed at baseline using the European Organisation for Research and Treatment of Cancer core questionnaire (EORTC QLQ-C30, version 3.0) (29) and the head-and-neck specific module (EORTC QLQ-H&N35) (30). Both questionnaires were self-administered and used a one-week recall period. Validated Spanish versions were used (31). Scale scores were calculated according to the EORTC scoring manual (32), applying a linear transformation to a 0–100 scale. For the QLQ-C30, higher scores indicate better functioning and global health status, whereas higher symptom-scale scores indicate greater symptom burden. For the QLQ-H&N35, higher scores reflect greater symptom burden or functional impairment. Missing items were handled in accordance with EORTC guidelines, with scale scores calculated when at least half of the items in a multi-item scale were completed. The internal consistency of the multi-item scales was assessed using Cronbach’s alpha (*α*) calculated from the item-level responses of the current sample. Reliability was adequate to excellent across all scales. For the QLQ-C30, alpha values were as follows, fatigue (*α* = 0.894), pain (*α* = 0.811), emotional functioning (*α* = 0.864), physical functioning (*α* = 0.701) and global health status (*α* = 0.932). For the QLQ-H&N35, values were pain (*α* = 0.842), swallowing (*α* = 0.854), senses/speech (*α* = 0.762) and social eating (*α* = 0.887).

### 2.4 Statistical analysis, sample size justification, and assessment of model adequacy

The distribution of continuous variables was assessed using the Shapiro–Wilk test. Variables not meeting normality assumptions (*p* < 0.05) are presented as median and interquartile range (IQR, 25th–75th percentile), whereas categorical variables are expressed as counts and percentages.

Comparisons of categorical variables between patients with GLIM-defined moderate-to-severe malnutrition and normonutrition patients were performed using the Chi-square test with Yates’ continuity correction. Comparisons of continuous biomarker variables between the two nutritional groups were performed using the non-parametric Mann–Whitney *U* test, given the non-normal distribution of circulating biomarker concentrations. The Mann–Whitney *U* test was also applied for group comparisons of EORTC QLQ-C30 and QLQ-H&N35 scores according to GLIM-defined nutritional status and according to anxiety status defined by the HADS-A subscale. To account for multiple testing in univariate analyses, *p*-values were adjusted using the Benjamini–Hochberg false discovery rate (FDR) procedure. Statistical significance after correction was defined as *q* < 0.05.

Circulating biomarker concentrations, including IL-6 and metabolic hormones, were natural log-transformed prior to regression analyses to reduce skewness and improve model stability. Regression coefficients therefore reflect the change in the outcome per unit increase in log-transformed biomarker levels.

Associations between continuous variables were evaluated using Spearman’s rank correlation coefficient (*ρ*), given the non-normal distribution of most biomarkers. All correlation analyses were adjusted for age and sex as potential confounders. Correlation results are presented as ρ coefficients with corresponding two-tailed *p*-values.

To identify independent predictors of pre-treatment health-related quality of life (HRQoL) outcomes, multivariable linear regression analyses were performed using ordinary least squares (OLS) estimation. Variables included in the models were selected based on clinical plausibility and variables showing at least trend-level associations (*p* < 0.10) in prior univariate analyses. Model fit was assessed using the adjusted *R*^2^ coefficient.

Independent predictors of pre-treatment malnutrition and clinically relevant anxiety were identified using multivariable binary logistic regression models. Variables included in each model were selected based on clinical plausibility and univariate associations. Model calibration was assessed using the Hosmer–Lemeshow goodness-of-fit test, and discrimination and explained variance were quantified using Nagelkerke’s *R*^2^. Results are reported as odds ratios (ORs) with 95% confidence intervals (CIs).

An *a priori* sample size calculation indicated that a minimum of 108 patients (approximately 43 malnourished and 65 normonutrition, assuming a 1.5:1 ratio) would be required to detect a moderate-to-large effect size (Cohen’s) in the primary association between anxiety (HADS-A) and malnutrition (GLIM) with 80% statistical power and a two-tailed alpha of 0.05. Due to strict clinical eligibility criteria and recruitment window constraints (2022–2024), recruitment was completed with 74 analyzed patients. For the observed effect size and achieved group allocation, this sample provided approximately 63% post-hoc power for the primary anxiety-malnutrition comparison.

Given this final sample size and the relatively limited number of outcome events (malnutrition: 40.54%, *n* = 30 events; and anxiety: 36.48%, *n* = 27 events), the events-per-variable (EPV) ratio for the multivariable logistic regression models was below the conventional threshold of 10. To minimize the risk of overfitting, predictor selection in each multivariable model was strictly restricted *a priori* based on clinical plausibility, prior literature, and univariate trend-level significance (*p* < 0.10), rather than relying on automated stepwise procedures. Finally, post-hoc statistical power, model calibration (Hosmer–Lemeshow test), and explained variance (Nagelkerke *R*^2^) were calculated for both multivariable logistic regression models to assess model adequacy (33–35).

A formal exploratory mediation analysis was conducted to evaluate whether anxiety (HADS-A score) statistically mediate the association between IL-6 and GLIM-defined pre-treatment malnutrition. The four causal pathways were estimated as follows: Path a (IL-6 → anxiety) was estimated by ordinary least squares (OLS) linear regression with anxiety score as the continuous outcome. Paths b, c, and c′ were estimated by binary logistic regression with malnutrition as the dichotomous outcome. The total effect (Path c) represents the association of IL-6 with malnutrition in the absence of the mediator; the direct effect (Path c′) represents the residual association after controlling for anxiety; and the indirect effect (Path a × b) quantifies the portion of the total effect transmitted through anxiety and was estimated using the product-of-coefficients approach (a × b) (36) and its statistical significance was assessed using bias-corrected bootstrap confidence intervals based on 5,000 resamples. Indirect effects are reported in their original, non-exponentiated scale, as recommended for mediation models combining linear and logistic components (36). The proportion of the total effect was mediated estimated as the ratio of the indirect to total effect. The proposed mediation model was based on an *a priori* psychoneuroimmunological framework derived from previous biological and clinical evidence suggesting that systemic inflammation may influence psychological distress and nutritional status. Interpretation of indirect effects therefore assumes temporal ordering and the absence of substantial unmeasured confounding-assumptions that cannot be verified within a cross-sectional design. Therefore, this analysis is exploratory and hypothesis-generating only (37–39).

All statistical analyses were performed using IBM SPSS Statistics (version 29, IBM Corp., Armonk, NY, USA). All tests were two-tailed and the nominal significance threshold was set at *p* < 0.05 prior to multiple-testing correction.

## 3 Results

### 3.1 Pre-treatment demographic and clinical characteristics

A total of 74 patients with LAHNC were included. The study population was predominantly composed of men (74.32%), with a mean age of approximately 62 years (range 28–78), and 19% aged ≥70 years. The most frequent primary tumour site was the oropharynx (72%), followed by the larynx (9%), oral cavity (8%), hypopharynx (6%), and nasopharynx (5%). At diagnosis, 53% of patients had ECOG performance status 0 and 47% had ECOG 1–2. Regarding tumour burden, 23% presented with T4 disease, while 77% had T1–T3 tumours. Nodal involvement was present in the majority of patients, with 50% classified as N0–N1 and 50% as N2–N3 (Table 1).

**Table 1:** Univariate associations between clinical and psychological variables and GLIM-defined malnutrition.

Variable	GLIM normonutrition (*n* = 44)	GLIM malnutrition (*n* = 30)	Overall (*n* = 74)	*p-*value	*q-FDR*
Sex				0.666	0.814
Male	34 (77.3%)	21 (70.0%)	55 (74.3%)		
Female	10 (22.7%)	9 (30.0%)	19 (25.7%)		
Age				0.910	0.956
<70 years	36 (81.81%)	24 (80%)	60 (81.1%)		
≥70 years	8 (18.18%)	6 (20%)	14 (18.9%)		
ECOG status				0.273	0.635
0	26 (59.1%)	13 (43.3%)	39 (52.7%)		
≥1	18 (40.9%)	17 (56.7%)	35 (47.3%)		
HPV				0.044	0.322
Negative	19 (43.18%)	22 (73.3%)	41 (55.4%)		
Positive	25 (56.82%)	8 (26.7%)	33 (44.6%)		
T stage				0.999	0.999
T1-T3	34 (77.3%)	23 (76.7%)	57 (77.0%)		
T4	10 (22.7%)	7 (23.3%)	17 (23.0%)		
N stage				0.097	0.355
N0-N1	26 (59.09%)	11 (36.66%)	37 (50.0%)		
N2-N3	18 (40.90%)	19 (63.33%)	37 (50.0%)		
Active smoking				0.066	0.355
No	33 (75%)	16 (53.3%)	49 (66.2%)		
Yes	11 (25%)	14 (46.7%)	25 (33.8%)		
Social support				0.290	0.635
Lived alone	14 (31.81%)	5 (16.67%)	19 (25.7%)		
Lived with family/partner	30 (68.18%)	25 (83.33%)	55 (74.3%)		
HADS-A				0.081	0.304
No case	32 (72.7%)	15 (50.0%)	47 (63.5%)		
Case	12 (27.3%)	15 (50.0%)	27 (36.5%)		

Statistical significance was assessed using the Chi-square test with Yates’ continuity correction. Data are expressed as *n* (%). False discovery rate (FDR) correction was applied with a threshold of *q* < 0.05. ECOG, Eastern Cooperative Oncology Group; HPV, human papillomavirus; HADS-A, Hospital Anxiety and Depression Scale—Anxiety subscale.

Nineteen patients (25.67%) lived alone, whereas 55 (74.32%) lived with a partner or family. HPV-positive tumours accounted for 44.59% of cases, while 55.40% were HPV-negative. Active smoking was reported by 25 patients (33.8%), and 63 (85.1%) had a smoking history of at least 10 pack-years. During treatment, 35 patients (47.3%) developed grade 3 mucositis and 39 (52.7%) grade 1–2 mucositis. The mean fentanyl dose for pain control was 28.8 μg per dose (range 0–75) (Table 1).

Pre-treatment malnutrition, defined by GLIM criteria, was identified in 30 patients (40.5%), while 44 (59.5%) were classified as normonutrition. Among the 30 GLIM-malnourished patients, 5 (16.7%) fulfilled the low-BMI phenotypic criterion, 4 (13.3%) met the diagnosis on the basis of unintentional weight loss, and 6 (20.0%) on the basis of reduced FFMI. Clinically relevant anxiety (HADS-A ≥ 8) was observed in 27 patients (36.5%) (Table 1).

### 3.2 Pre-treatment nutritional status and biomarker associations

Pre-treatment BMI was significantly lower in patients with malnutrition compared with normonutrition individuals (21.4 [18.8–23.2] vs. 27.2 [24.1–30.5] kg/m^2^; q < 0.001).

Pre-treatment anxiety was more frequent in malnutrition patients compared with normonutrition individuals (50.0% vs. 27.3%), although this difference did not reach statistical significance after FDR correction (*q* = 0.304) (Table 1).

Regarding HPV status, HPV-positive patients were more frequently normonutrition compared with HPV-negative patients (56.82% vs. 26.7%; *p* = 0.044; *q* = 0.322). In contrast, patients with HPV-negative tumours showed a more balanced distribution between normonutrition and malnutrition status. Finally, patients with N0–N1 stage were more frequently normonutrition compared with those with N2–N3 disease (59.1% vs. 36.7%; *q* = 0.355) (Table 1).

Among circulating inflammatory and metabolic/appetite-regulating biomarkers, patients with malnutrition exhibited significantly higher levels of GIP (27.05 [17.55–47.55] vs. 12.95 [9.22–22.20] pg./mL; *q* = 0.003) and IL-6 (28.94 [14.50–56.12] vs. 10.35 [5.42–21.80] pg./mL; *q* = 0.009). Other metabolic and inflammatory markers did not remain significant after FDR correction (Table 2).

**Table 2:** Univariate associations between metabolic/appetite-regulating and inflammatory biomarkers and GLIM-defined malnutrition.

Biomarker	GLIM normonutrition (*n* = 44)	GLIM malnutrition (*n* = 30)	*p-*value	*q-FDR*
Metabolic/appetite-regulating biomarkers				
PYY (pg/mL)	64.10 [40.45–141.20]	45.80 [32.70–70.08]	0.139	0.397
PP (pg/mL)	101.30 [64.28–155.90]	102.80 [74.08–198.12]	0.575	0.736
Ghrelin (pg/mL)	345.02 [227.13–740.0]	420.21 [262.12–1118.10]	0.384	0.704
GIP (pg/mL)	12.95 [9.22–22.20]	27.05 [17.55–47.55]	<0.001	0.003
GLP-1 (pg/mL)	120.65 [98.15–154.43]	115.80 [88.80–145.80]	0.597	0.736
Insulin (μIU/mL)	4.85 [3.23–8.10]	3.61 [2.11–5.93]	0.038	0.076
Leptin (pg/mL)	2594.9 [1493–3,684]	1872.3 [408–3,824]	0.144	0.397
C-peptide (pmol/L)	1114.45 [760.27–1564.00]	1207.85 [974.68–1591.75]	0.241	0.675
Inflammatory biomarkers				
IL-1 α (pg/mL)	17.70 [4.60–35.50]	7.50 [4.50–39.30]	0.880	0.948
IL-6 (pg/mL)	10.35 [5.42–21.80]	28.94 [14.50–56.12]	<0.001	0.009
IL-8 (pg/mL)	13.55 [9.93–19.65]	14.80 [11.62–19.30]	0.317	0.635
IL-10 (pg/mL)	6.80 [3.90–11.20]	5.95 [3.20–9.80]	0.384	0.512
TNF-α (pg/mL)	39.85 [27.93–46.45]	36.65 [29.82–41.25]	0.631	0.814
IFN-α2 (pg/mL)	69.60 [25.40–102.70]	68.10 [27.60–167.05]	0.990	0.990

Group comparisons were performed using the Mann–Whitney *U* test. Data are expressed as median [IQR]. FDR correction was applied at *q* < 0.05. GIP, glucose-dependent insulinotropic polypeptide; GLP-1, glucagon-like peptide-1; PP, pancreatic polypeptide; PYY, peptide YY; IL, interleukin; TNF-α, tumour necrosis factor-α; IFN-α2, interferon-α2.

### 3.3 Correlation between inflammatory and metabolic biomarkers

Spearman correlation analyses adjusted for age and sex revealed significant associations between pro-inflammatory cytokines and metabolic/appetite-regulating hormones (Table 3). IL-1*α* showed a strong positive correlation with ghrelin (*ρ* = 0.566, *p* = 0.001), while IL-6 was positively correlated with ghrelin (*ρ* = 0.339, *p* = 0.003). TNF-α was positively associated with both GIP (*ρ* = 0.324, *p* = 0.005) and insulin (*ρ* = 0.301, *p* = 0.011). In contrast, IL-8 showed an inverse correlation with leptin levels (*ρ* = −0.340, *p* = 0.003) (Table 3).

**Table 3:** Correlation between inflammatory cytokines and metabolic/appetite-regulating biomarkers in patients with head and neck cancer.

Inflammatory biomarkers	Metabolic/Appetite-regulating biomarkers	Rho (*ρ*)	*p-*value*
IL-1α	Ghrelin	0.566	0.001
IL-6	Ghrelin	0.339	0.003
TNF-α	GIP	0.324	0.005
	Insulin	0.301	0.011
IL-8	Leptin	−0.340	0.003

Correlation coefficients (Spearman’s Rho) were adjusted for age and sex as potential confounders. *p < 0.05 was considered as significant.

### 3.4 Health-related quality of life according to nutritional and anxiety status

Pre-treatment HRQoL, assessed using the EORTC QLQ-C30 and QLQ-H&N35 questionnaires, was significantly impaired in patients with GLIM-defined moderate-to-severe malnutrition. Compared with normonutrition individuals, malnutrition patients exhibited lower physical and emotional functioning together with a higher symptom burden, including fatigue and pain (all *q* ≤ 0.05). In addition, they reported significantly worse head-and-neck–specific symptoms, particularly pain, swallowing difficulties, sensory problems, and social eating limitations (all *q* ≤ 0.02) (Table 4). Pre-treatment anxiety was also strongly associated with poorer HRQoL. Patients with anxiety demonstrated lower physical functioning, emotional functioning, and global health status (*q* = 0.009, *q* = 0.008, and *q* = 0.046, respectively), together with higher head-and-neck pain, fatigue, sensory problems and more severe swallowing impairment (*q* = 0.042, *q* = 0.043, *q* = 0.045 and *q* = 0.043) (Table 5).

**Table 4:** Univariate associations between EORTC QLQ variables and GLIM-defined malnutrition.

Scale	GLIM Normonutrition (*n* = 44)	GLIM Malnutrition (*n* = 30)	*p-*value	*q-FDR*
QLQ-C30 Physical functioning	93.4 [83.3–100.0]	86.1 [80.0–96.7]	0.002	0.004
QLQ-C30 Fatigue	12.2 [0.0–16.7]	25.0 [11.1–38.9]	0.001	0.003
QLQ-C30 Pain	19.8 [0.0–33.3]	40.2 [16.7–66.7]	0.011	0.017
QLQ-C30 Emotional functioning	85.7 [75.0–100.0]	67.8 [41.7–91.7]	0.019	0.024
QLQ-C30 Global Health Status	70.4 [58.3–83.3]	59.8 [41.7–83.3]	0.080	0.195
QLQ-H&N35 Pain	27.9 [8.3–41.7]	55.2 [33.3–75.0]	0.001	0.003
QLQ-H&N35 swallowing	12.4 [0.0–16.7]	28.7 [8.3–44.4]	0.002	0.004
QLQ-H&N35 Social eating	14.0 [0.0–16.7]	47.7 [25.0–75.0]	<0.001	0.003
QLQ-H&N35 Sensory problems	8.1 [0.0–16.7]	21.9 [0.0–33.3]	0.025	0.028

Statistical significance was determined via the Mann–Whitney *U* test. Data are expressed as median [IQR]. FDR correction was applied at *q* < 0.05. EORTC, European Organisation for Research and Treatment of Cancer; QLQ, Quality of Life Questionnaire; GLIM, Global Leadership Initiative on Malnutrition.

**Table 5:** Univariate associations between EORTC QLQ variables and anxiety status.

Scale	No anxiety (HADS-A < 8, *n* = 47)	Anxiety (HADS-A ≥ 8, *n* = 27)	*p-*value	*q-FDR*
QLQ-C30 Physical functioning	100.0 [91.5–100.0]	83.0 [83.0–100.0]	0.002	0.009
QLQ-C30 Fatigue	7.4 [3.7–20.4]	22.2 [11.1–33.3]	0.024	0.043
QLQ-C30 Pain	16.7 [0.0–33.3]	33.3 [0.0–66.7]	0.185	0.208
QLQ-C30 Emotional functioning	100.0 [66.7–100.0]	66.7 [33.3–66.7]	<0.001	0.008
QLQ-C30 Global Health Status	75.0 [58.3–83.3]	66.7 [33.3–83.3]	0.036	0.046
QLQ-H&N35 Pain	33.3 [16.7–41.7]	50.0 [16.7–83.3]	0.014	0.042
QLQ-H&N35 Swallowing	7.7 [0.0–16.7]	16.7 [12.7–33.3]	0.019	0.043
QLQ-H&N35 Sensory problems	6.1 [0.0–15.2]	16.7 [0.0–33.3]	0.030	0.045
QLQ-H&N35 Social Eating	16.3 [0.0–32.5]	33.6 [0.0–68.5]	0.059	0.066

Statistical significance was determined via the Mann–Whitney *U* test. Data are expressed as median [IQR]. FDR correction was applied at *q* < 0.05. HADS-A, Hospital Anxiety and Depression Scale—Anxiety subscale; EORTC, European Organisation for Research and Treatment of Cancer.

### 3.5 Multivariable predictors of HRQoL

In multivariable linear regression models, poorer physical functioning was independently associated with worse ECOG performance status (*B* = −11.43, *β* = −0.52, *p* < 0.001) and the presence of anxiety (*B* = −0.90, *β* = −0.26, *p* = 0.048), while IL-6 levels showed a trend toward significance (*B* = −7.436, *β* = −0.20, *p* = 0.065). Swallowing impairment was independently predicted by malnutrition (*B* = 8.98, *β* = 0.38, *p* = 0.005) and anxiety (*B* = 1.39, *β* = 0.29, *p* = 0.033). Head-and-neck pain was independently related to social eating difficulties (*B* = 0.47, *β* = 0.52, p < 0.001), swallowing impairment (*B* = 0.42, *β* = 0.34, *p* = 0.003), and lower emotional functioning (*B* = −0.28, *β* = −0.30, *p* = 0.003). Finally, global health status was independently associated with greater social eating difficulties (*B* = −0.26, *β* = −0.27, *p* = 0.010), higher pain burden (*B* = −0.27, *β* = −0.29, *p* = 0.007), and anxiety (*B* = −1.29, *β* = −0.25, *p* = 0.043) (Table 6).

**Table 6:** Multivariable linear regression models for baseline health-related quality of life outcomes.

Outcome	Predictor	Adjusted *R*^2^	*B* (SE)	*β*	*p-*value*
QLQ-C30 Physical functioning	ECOG	0.275	−11.43 (2.85)	−0.52	<0.001
	IL-6		−7.436 (3.968)	−0.20	0.065
	Anxiety (HADS-A)		−0.90 (0.44)	−0.26	0.048
QLQ-C30 Global health status	Social eating (H&N35)	0.465	−0.26 (0.10)	−0.27	0.010
	Pain (C30)		−0.27 (0.09)	−0.29	0.007
	Anxiety (HADS-A)		−1.29 (0.62)	−0.25	0.043
QLQ-H&N35 Swallowing	GLIM	0.210	8.98 (3.08)	0.38	0.005
	Anxiety (HADS-A)		1.39 (0.63)	0.29	0.033
QLQ-H&N35 Pain	Social eating (H&N35)	0.666	0.47 (0.09)	0.52	<0.001
	Swallowing (H&N35)		0.42 (0.13)	0.34	0.003
	Emotional functioning (C30)		−0.28 (0.09)	−0.30	0.003
	ECOG		2.15 (1.53)	0.12	0.161

Analyses were performed using ordinary least squares (OLS) to identify independent predictors. Adjusted *R*^2^ values indicate model fit. For QLQ-C30 functioning scales, higher scores indicate better functioning; for symptom scales and QLQ-H&N35 scales, higher scores indicate greater symptom burden. *p < 0.05 was considered as significant. GLIM, Global Leadership Initiative on Malnutrition; HRQoL, health-related quality of life; ECOG, Eastern Cooperative Oncology Group.

### 3.6 Independent predictors of malnutrition and anxiety

In multivariable logistic regression analyses (Table 7), elevated IL-6 (OR 6.16, 95% CI 1.35–28.18; *p* = 0.019) and lower BMI (OR 0.83, 95% CI 0.72–0.97; *p* = 0.023) were independently associated with pre-treatment malnutrition. For anxiety, elevated IL-6 emerged as the only significant independent predictor (OR 11.23, 95% CI 2.09–60.40; *p* = 0.005). Female sex showed a trend toward greater anxiety risk (OR 3.91, 95% CI 0.91–16.74; *p* = 0.066), while other clinical variables including age, ECOG status, tumour stage, HPV status, and smoking did not reach statistical significance in either model (Table 7).

**Table 7:** Multivariable logistic regression analyses identifying independent predictors of pre-treatment malnutrition and anxiety.

Outcome	Predictor	OR (95% CI)	*p-*value*
Pre-treatment Malnutrition Nagelkerke *R*^2^:0.417 Hosmer–Lemeshow (p): 0.915	IL-6	6.16 [1.35–28.18]	0.019
	BMI	0.83 [0.72–0.97]	0.023
	Age	1.02 [0.95–1.11]	0.494
	Sex	2.94 [0.64–13.42]	0.164
	ECOG status	2.36 [0.69–8.08]	0.171
	Tumor stage	1.21 [0.84–1.72]	0.294
	HPV Status	1.01 [0.18–5.66]	0.991
	Smoking Status	1.79 [0.49–6.47]	0.375
Anxiety Nagelkerke *R*^2^: 0.354 Hosmer–Lemeshow (p): 0.957	IL-6	11.23 [2.09–60.39]	0.005
	BMI	1.07 [0.93–1.24]	0.315
	Age	0.93 [0.86–1.01]	0.1
	Sex	3.91 [0.91–16.74]	0.066
	ECOG Status	0.99 [0.30–3.28]	0.993
	Tumor Stage	1.15 [0.82–1.62]	0.4
	HPV Status	0.58 [0.09–3.44]	0.556
	Smoking Status	0.65 [0.17–2.52]	0.536

Nagelkerke *R*^2^ was used to quantify explained variance, and model calibration was assessed via the Hosmer–Lemeshow test. *p < 0.05 was considered as significant. OR, odds ratio; CI, confidence interval; BMI, Body Mass Index; HPV, human papillomavirus; ECOG, Eastern Cooperative Oncology Group.

Multivariable logistic regression models demonstrated high explanatory power. The model for anxiety reached a post-hoc statistical power of 87.1%, corresponding to the observed effect of IL-6 (*p* = 0.005). For the malnutrition model, the statistical power was 65.1% (*p* = 0.019). Both models showed good calibration (Hosmer–Lemeshow *p* > 0.05) and substantial explanatory capacity, with Nagelkerke *R*^2^ of 0.354 and 0.417 for anxiety and malnutrition, respectively. Significant predictors exhibited substantial biological relevance: IL-6 presented a large effect size in both models (OR > 6.0), while BMI acted as a consistent protective factor against malnutrition (OR = 0.83 per unit increase) (Table 7).

### 3.7 Mediation analysis of the IL-6–anxiety–malnutrition pathway

**Figure 1:**
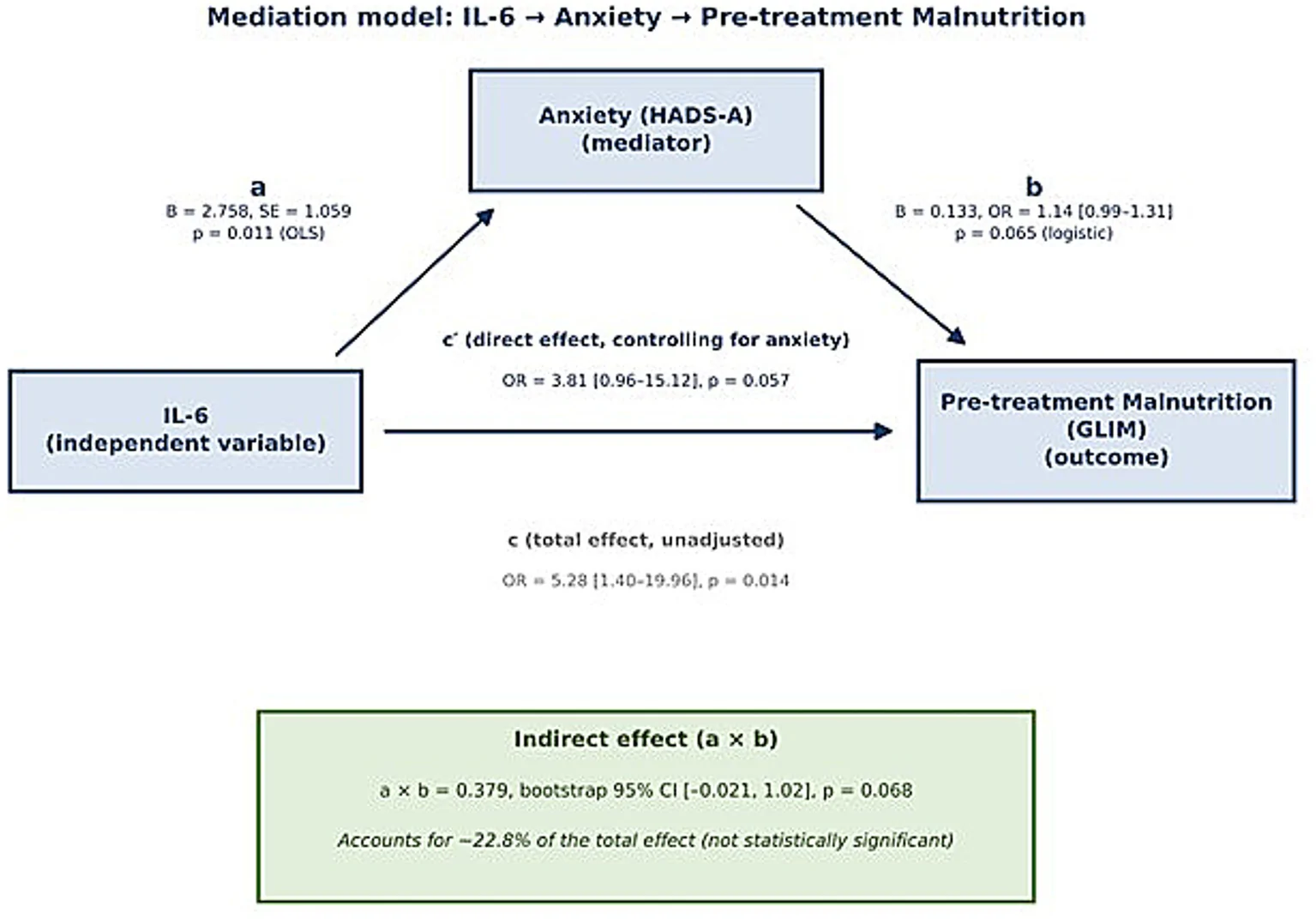
Mediation model of the association between IL-6, anxiety, and pre-treatment malnutrition in patients with LAHNC. Path *a*: IL-6 → anxiety (OLS regression). Path *b*: Anxiety → malnutrition, controlling for IL-6 (logistic regression). Path *c*: Total, unadjusted effect of IL-6 on malnutrition. Path *c′*: Direct effect of IL-6 on malnutrition after adjusting for anxiety. The indirect effect (*a* × *b*) represents the portion of the IL-6-malnutrition association potentially transmitted through anxiety, estimated via bias-corrected bootstrap resampling (5,000 iterations).

To explore whether anxiety mediated the association between IL-6 and pre-treatment malnutrition, we conducted a bootstrap mediation analysis (Figure 1). The total effect of IL-6 on pre-treatment malnutrition was significant (OR = 5.28, *p* = 0.014), corresponding to a more than fivefold increase in odds. After inclusion of anxiety as a mediator, the direct effect of IL-6 was attenuated and no longer reached strict statistical significance (OR = 3.81, *p* = 0.057). The indirect effect through anxiety suggested a possible partial mediation pattern, accounting for approximately 22.8% of the total effect; however, this did not reach statistical significance (*p* = 0.068). The bootstrap confidence interval crossed zero, indicating that the indirect effect did not reach statistical significance and should therefore be interpreted cautiously (Table 8).

**Table 8:** Mediation analysis of the association between IL-6, anxiety, and pre-treatment malnutrition.

Path	*B*	SE	OR [95%CI]	*p-*value*	Interpretation
a (IL-6 → anxiety)	2.758	1.059	—	0.011	Higher IL-6 is significantly associated with greater anxiety score.
b (anxiety→ malnutrition, controlling for IL-6)	0.133	0.072	1.14 [0.99–1.31]	0.065	Higher anxiety is associated with increased odds of malnutrition, although this did not reach statistical significance.
c (total effect: IL-6 → malnutrition)	1.664	0.679	5.28 [1.40–19.96]	0.014	IL-6 is associated with a 5.28-fold increase in the odds of pre-treatment malnutrition.
C’ (direct effect: IL-6 → malnutrition, controlling for anxiety)	1.337	0.730	3.81 [0.96–15.12]	0.057	The association between IL-6 and malnutrition is attenuated after adjustment for anxiety, consistent with partial mediation.
A × b (indirect effect: IL-6 → anxiety → malnutrition)	0.379	0.269	Bootstrap 95% CI: −0.021-1.02.	0.068	The indirect effect was positive but did not reach statistical significance (bootstrap CI crosses zero), providing only suggestive evidence of partial mediation. Anxiety accounted for approximately 22.8% of the total effect.

Path a estimated by OLS regression; paths b, c, and c’ were estimated by binary logistic regression. Indirect effect significance was determined using bias-corrected bootstrap resampling with 5,000 iterations. *p < 0.05 was considered as significant. B, unstandardized coefficient; SE, standard error; OR, odds ratio; CI, confidence interval.

## 4 Discussion

This study investigated the interplay between psychological distress, systemic inflammation, and nutritional status in patients with LAHNC treated with definitive radiochemotherapy. Our findings show that elevated IL-6 levels were independently associated with anxiety, and represented the only independent predictor of anxiety in multivariable analysis. In addition, elevated IL-6 and lower BMI were independently associated with pre-treatment malnutrition. Exploratory mediation analysis suggested a possible indirect pathway linking IL-6 with malnutrition through anxiety, however, the indirect effect did not reach statistical significance and should therefore be interpreted cautiously. Overall, these findings are consistent with a psychoneuroimmunological framework linking systemic inflammation, emotional distress, and nutritional status, although causal relationships cannot be inferred.

These findings are broadly consistent with preclinical evidence linking social stress to increased IL-6 expression and IL-1-mediated neuroinflammation associated with anxiety-like behaviour (40), and with clinical data showing higher circulating and cerebrospinal IL-6 in individuals with anxiety disorders compared with healthy controls, independent of depressive symptoms (41, 42). Cerebrospinal IL-6 has also been associated with trait anxiety and altered cerebral blood flow in emotion-processing regions, supporting a plausible biological link between IL-6 and anxiety (42).

In our study population, the prevalence of clinically relevant anxiety was higher among patients with GLIM-defined moderate-to-severe malnutrition compared with normonutrition individuals (50% vs. 27.3%); although this association did not remain statistically significant after FDR correction, and the mediation indirect effect did not reach statistical significance. These findings therefore suggest a possible relationship between psychological distress and nutritional status rather than robust evidence of a mediating role for anxiety. They are broadly in line with previous reports associating psychological distress in head and neck cancer with weight loss, impaired nutritional status, reduced treatment tolerance and increased toxicity (17, 43), and with studies linking emotional distress to malnutrition risk and adverse outcomes through inflammation-related pathways, including a nearly twofold higher risk of clinically relevant anxiety among malnourished cancer patients (44, 45).

Mechanistically, chronic psychological stress may activate the hypothalamic–pituitary–adrenal (HPA) axis and sympathetic nervous system, promoting release of pro-inflammatory cytokines such as IL-6 and TNF-α, which have been implicated in cancer-associated cachexia, anorexia, and immune dysregulation (46). Cancer-related anxiety has been associated with elevated IL-6, TNF-α and CRP in newly diagnosed head and neck cancer patients, with IL-6 proposed as a bridging node between psychoneurological and inflammatory symptom clusters, suggesting that anxiety may partly reflect tumour-host inflammatory interactions rather than a purely reactive psychological response (47).

Consistent with this framework, our exploratory mediation analysis suggested a possible indirect pathway linking IL-6 with pre-treatment malnutrition through anxiety, although the indirect effect did not reach statistical significance and the attenuation of the direct IL-6-malnutrition association after including anxiety does not constitute definitive evidence of mediation. These findings are, at most, compatible with the plausibility of anxiety as a contributing factor within a broader psychoneuroimmunological context, given that IL-6 has been linked to cancer-related cachexia, fatigue and anxiety symptoms (46, 48, 49), possibly via HPA-axis activation and gut-brain signalling (12, 50).

In addition to IL-6, GIP was significantly elevated in patients with malnutrition. GIP is an incretin hormone involved in adipogenesis, energy partitioning and central appetite regulation (51). In the context of cancer-related malnutrition, elevated GIP may reflect a dysregulated incretin response to impaired intake or altered gut permeability, although the mechanistic implications remain uncertain and warrant further investigation.

The observed correlations between pro-inflammatory cytokines (IL-1*α*, IL-6, IL-8, TNF-α) and gut-derived hormones (ghrelin, GIP, insulin, leptin) are consistent with an integrated neuroendocrine-immune network potentially linking systemic inflammation to disruptions in appetite-regulating hormones, providing a plausible biological substrate for nutritional decline in LAHNC. Interventions targeting IL-6 signalling (52), or non-pharmacological strategies such as structured physical exercise (through its myokine-mediated anti-inflammatory effects), may represent avenues worth exploring to mitigate systemic inflammation and preserve nutritional status (53).

HPV positivity was associated with lower rates of malnutrition in univariate analysis, although this association did not remain significant after FDR correction or in multivariable models. This finding aligns with previous reports indicating that HPV-related tumours often occur in younger patients with lower cumulative exposure to tobacco and alcohol, which may partly explain their more favourable nutritional and clinical profiles (54).

GLIM-defined malnutrition was consistently associated with impaired HRQoL across multiple domains and independently predicted swallowing impairment in multivariable analysis, consistent with previous evidence that GLIM-defined malnutrition is associated with worse HRQoL, particularly pain, swallowing difficulties and social eating, during and after treatment in advanced head and neck cancer (6, 55). The bidirectional relationship between nutritional deterioration and dysphagia is well recognised (56), reinforcing the value of integrating nutritional assessment with structured swallowing evaluation from treatment initiation to enable timely prehabilitation (57, 58).

The multidimensional structure of pre-treatment global HRQoL observed in our study population, driven by a convergence of pain, emotional distress, and social eating limitations, reflects the well-described biopsychosocial complexity of this population. Social eating restrictions are a particularly consequential domain given the relational and cultural significance of communal eating (59). Anxiety, as captured by the HADS, has been independently associated with impaired HRQoL and treatment tolerance, and has been reported more frequently among malnourished/cachectic patients at pre-treatment assessment, with lower bioelectrical impedance phase angle values, consistent with a link between anxiety and nutritional deterioration (60). Uncontrolled pain may further compound this picture by limiting oral intake and reinforcing social withdrawal and nutritional decline.

From a clinical perspective, these results support the systematic implementation of a multidisciplinary pre-treatment screening protocol integrating brief psychological distress assessment (HADS), standardised nutritional evaluation (GLIM), symptom monitoring, and structured swallowing screening. Integrated psycho-oncological support, dietary counselling, and proactive speech and language therapy may improve treatment tolerance, adherence and patient-reported outcomes during RCT (6, 59), and psychoneuroimmunology-informed strategies addressing HPA-axis dysregulation and inflammatory burden could further support a mechanistically coherent, personalised care model (60).

Several limitations should be acknowledged. First, the cross-sectional design precludes causal inference, and reverse causation between IL-6, anxiety, and pre-treatment malnutrition cannot be excluded. Second, although an *a priori* sample size calculation was performed for the primary anxiety-malnutrition comparison (*n* = 108), the final recruited sample (*n* = 74) fell short of this target owing to feasibility constraints during the study period, yielding approximately 63% power for this comparison; furthermore, the sample of 74 patients yielded an events-per-variable ratio below the recommended threshold of 10, which may limit coefficient stability and increase overfitting risk; the findings should therefore be considered exploratory and hypothesis-generating, pending validation in larger, adequately powered cohorts. Third, single-centre recruitment limits generalisability to other settings, tumour subsites, and populations with different nutritional care protocols. Fourth, the mediation analysis assumes the absence of unmeasured confounding in the IL-6 → anxiety → malnutrition pathway, although patients with major chronic inflammatory/metabolic comorbidities and prior enteral nutritional support were excluded, residual confounding due to other factors such as pre-existing psychiatric conditions, corticosteroid use, or socioeconomic status cannot be fully excluded. Fifth, the single pre-treatment HRQoL assessment precludes characterisation of quality-of-life trajectories throughout treatment or attribution of impairments to specific disease, psychological, or nutritional factors. Finally, HADS-based anxiety classification, although validated in oncological populations, is subject to response bias and does not constitute a formal psychiatric diagnosis.

Despite these limitations, this study has several notable strengths, including the simultaneous quantification of a broad panel of inflammatory cytokines and gut-derived hormones in a well-characterised, consecutively recruited study population, the use of validated nutritional (GLIM), psychological distress (HADS), and health-related quality of life (QLQ-C30/HN35); and the application of formal bootstrap mediation analysis to explore a psychoneuroimmunological hypothesis poorly examined in head and neck cancer. The use of HADS-A further strengthens the interpretation of these findings because the instrument minimizes contamination by somatic symptoms frequently associated with cancer and its treatment, allowing a more specific assessment of anxiety-related psychological distress.

## 5 Conclusion

In conclusion, IL-6 emerged as a key factor associated with both anxiety and pre-treatment malnutrition in patients with LAHNC, while lower BMI was an additional independent predictor of malnutrition. Although anxiety did not reach independent significance in the fully adjusted malnutrition model, exploratory bootstrap mediation analysis suggested a possible indirect association between IL-6 and malnutrition through anxiety that warrants confirmation in longitudinal studies. Overall, these findings are consistent with a psychoneuroimmunological framework linking systemic inflammation, emotional distress, and nutritional status, although causal relationships cannot be established. These findings should therefore be considered hypothesis-generating until replicated in adequately powered prospective cohorts. Integrative, multidisciplinary care strategies addressing both biological and psychological factors may help optimise nutritional and clinical outcomes in this vulnerable population.

## Data Availability

The raw data supporting the conclusions of this article will be made available by the authors, without undue reservation.

## Glossary

BIA: Bioelectrical impedance analysis
BMI: Body Mass Index
CI: Confidence interval
CRP: C-reactive protein
CV: Coefficient of variation
ECOG: Eastern Cooperative Oncology Group
EORTC: European Organisation for Research and Treatment of Cancer
EPV: Events per variable
FDR: False discovery rate
FFM: Fat-free mass
FFMI: Fat-Free Mass Index
GIP: Glucose-dependent insulinotropic polypeptide
GLIM: Global leadership initiative on malnutrition
GLP-1: Glucagon-like peptide-1
HADS: Hospital Anxiety and Depression Scale
HADS-A: Hospital Anxiety and Depression Scale–Anxiety Subscale
HPA: Hypothalamic–pituitary–adrenal
HPV: Human papillomavirus
HRQoL: Health-related quality of life
IFN-*α*2: Interferon alpha-2
IL-1α: Interleukin-1 alpha
IL-6: Interleukin-6
IL-8: Interleukin-8
IL-10: Interleukin-10
IQR: Interquartile range
LAHNC: Locally advanced head and neck cancer
OLS: Ordinary least squares
OR: Odds ratio
PNI: Psychoneuroimmunology
PP: Pancreatic polypeptide
PYY: Peptide YY
QLQ-C30: Quality of Life Questionnaire-Core 30
QLQ-H&N35: Quality of Life Questionnaire Head and Neck Module 35
RCT: Radiochemotherapy
SPSS: Statistical Package for the Social Sciences
TNF-α: Tumor necrosis factor alpha
